# Evaluating the Adequacy of YouTube Videos for Learning Laparoscopic Right Hemicolectomy With Complete Mesocolic Excision

**DOI:** 10.7759/cureus.65760

**Published:** 2024-07-30

**Authors:** Adem Şentürk, Tarik Harmantepe

**Affiliations:** 1 Surgical Oncology, Sakarya University Training and Research Hospital, Sakarya, TUR; 2 Gastroenterology Surgery, Sakarya University Training and Research Hospital, Sakarya, TUR

**Keywords:** unreliable, total mesocolic excision, reliable, laparoscopic right hemicolectomy, youtube videos

## Abstract

Aim: Complete mesocolic excision (CME) is becoming an oncological surgical principle day by day for right hemicolectomy. However, the procedure is technically difficult and carries a higher risk of complications than open surgery. In this study, the adequacy of YouTube videos that facilitate education for laparoscopic right hemicolectomy with complete mesocolic excision (LRHCME) was investigated.

Methods: In July 2024, in the search bar of the YouTube platform, the term "laparoscopic right hemicolectomy complete mesocolic excision" was searched. The first 100 videos in each search were evaluated. Animations, advertisements, lectures, non-surgical videos (pre-surgery, post-surgery vlog, etc.), and non-English videos were excluded from the study. Steps identified in the Delphi consensus were used to determine the reliability of the videos. The quality of the videos was measured using the Global Quality Scale (GQS) and the modified DISCERN score.

Results: Seventy videos were included in the evaluation. While 28 (40%) of these videos were classified as reliable, 42 (60%) were not found reliable. In reliable videos, video description, HD resolution, GQS, modified DISCERN, and duration were significantly higher (p-value <0.001, 0.012, <0.001, <0.001, 0.041 respectively). Reliable videos had a better rank than unreliable videos (p=0.046).

Conclusion: When evaluated according to Delphi consensus, the most of LRHCME videos on the YouTube platform were unreliable. We conclude that YouTube alone is insufficient for learning LRHCME without a professional instructor.

## Introduction

Complete oncological resection affects recurrence and survival. Complete mesocolic excision (CME), first described by Hohenberger, is based on embryological planes [1]. With CME, more lymph nodes are resected. Accordingly, the recurrence rate decreases and disease-free survival increases [2,3]. It can be applied to CME, right hemicolectomy, and all other colon surgeries. It has become standard along with colon surgery in many centers. However, there is an increased complication rate with CME compared to traditional colon surgery [4]. Superior mesenteric vein and other organ injuries are significantly increased in CME compared to the traditional approach [5]. It requires good anatomy knowledge and surgical experience to minimize complications. The learning curve of the first assistant for laparoscopic right hemicolectomy with CME (LRHCME) was calculated as a minimum of 32 cases [6].

Studies have shown that video education can improve surgical and clinical skills using teaching modules for medical professionals [7,8]. As a matter of fact, experts improve their skills by sharing video recordings at scientific congresses. Nowadays, books and magazines are now being replaced by online videos for accessing information. Because it is more up-to-date and easier to access, the popularity of video in education is increasing. YouTube is the most popular online video platform with more than one billion users and unlimited video sharing. Just as there are all kinds of video sharing, there are also medical information and even surgical videos. It is the platform frequently used by trainers today to improve their skills [9,10]. This situation was achieved very quickly during the COVID-19 pandemic [11]. However, the reliability of videos on YouTube is controversial. Many studies in the literature have found that YouTube videos are insufficient for learning surgery [12-15]. Because videos are uploaded also by people who are not educators without any educational purpose.

This study aims to evaluate and determine the educational value of publicly available LRHCME videos on YouTube in terms of surgical education.

## Materials and methods

**Figure 1:**
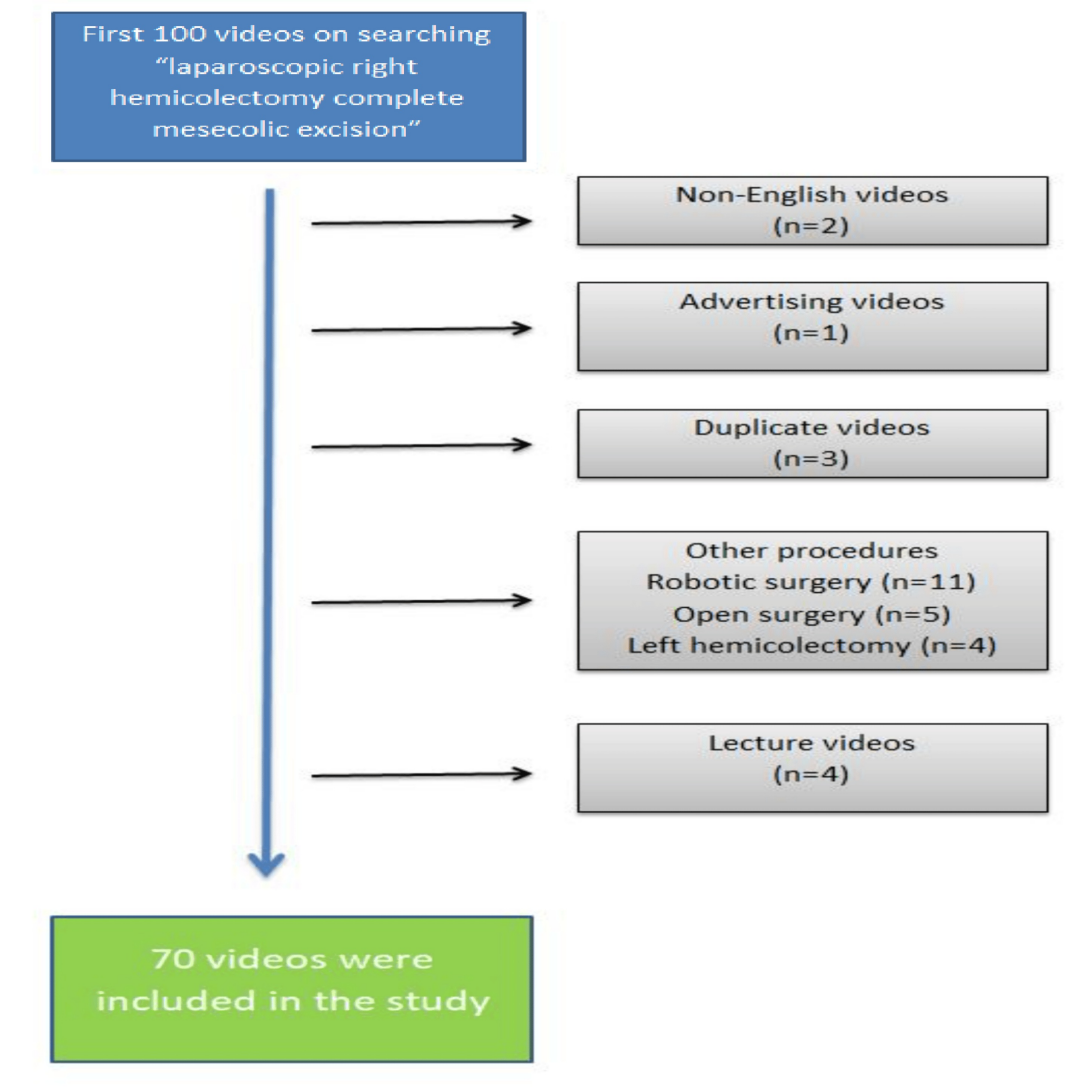
Videos excluded from the study

In July 2024, the term "laparoscopic right hemicolectomy complete mesocolic excision" was searched in the search bar of the YouTube platform. The sort setting remained relevant. The first 100 videos were evaluated in the search. Lectures, animations, advertisements, videos other than English, open surgery, robotic surgery, and non-operative videos (pre-operative, post-operative vlog, etc.) were excluded from the study (Figure 1).

The videos were evaluated by two surgeons experienced in oncological colon surgery. The reliability of the surgery in the videos was assessed using the key steps of the Delphi consensus (Table 1). The stages of pre-operative preparation, setup, and completion of the surgery are absent in almost all videos. Therefore, by excluding these steps, the videos were defined as unreliable when any of the other steps were not shown, and reliable when all of them were shown. The year of upload, approach (lateral to medial (LatMed), medial to lateral (MedLat)) resolution, number of comments, number of views, ranking, likes, and dislikes of the videos were also evaluated.

**Table 1:** Laparoscopic right hemicolectomy with complete mesocolic excision key steps SMV superior mesenteric vein

Key steps
Preoperative preparation
Setup of laparoscopy
Left paraumbilical 12 mm port using open technique or Veress needle
(anti)Trendelenburg depending on phase of the procedure
Dissection
Release of the omentum from the transverse colon
Traction of the cecum and detection of ileocolic vessels
Cutting the peritoneum parallel to the ileocolic vessels
Blunt dissection of Toldt's fascia towards the lateral abdominal wall
Continue dissection above the duodenum and until the gastrocolic trunk enters the SMV.
Closure of ileocolic vessels
Division of the lateral peritoneal reflection of the ascending colon along Toldt's white line
Mobilization of the hepatic flexure
Division of the right mesocolon, including the right colic artery and the right branch of the middle colic artery
Division of the mesentery of the ileum
Intracorporeal or extracorporeal incision of the terminal ileum and transverse colon with a linear stapler
Minilaparotomy, specimen removal and anastomosis
Anastomosis (intra or extracorporeal) according to the surgeon's preference
Closure of extraction zone and port sites 10 mm or greater
Completion of the operation

The quality of educational content in the videos was measured using the Global Quality Scale (GQS). A modified DISCERN score was also applied to examine the reliability and quality dimensions of the videos (Table 2).

**Table 2:** Global Quality Scale (GQS) and modified DISCERN scoring

The Global Quality Scale (GQS)	Scores from 1 to 5	The modified DISCERN	Score for each Yes: 1 Point No: 0 Point
The video exhibits poor quality, lacks a coherent structure, lacks essential information, and provides minimal patient benefit.	1	Does the video demonstrate clarity, brevity, and understandability?	Yes/No
The video is generally of below-average quality and lacks proper presentation. While some information is included, many important aspects are missing, resulting in limited patient value.	2	Is the video based on reliable sources of information? (e.g., quotes from broadcasts featuring expert speakers)	Yes/No
Although the quality of the video is average, its flow could be improved. While some critical information is adequately covered, other aspects are inadequately protected, resulting in moderate usefulness for patients.	3	Does the information provided maintain a balanced and unbiased perspective?	Yes/No
The video exhibits commendable quality with a smooth flow. It effectively covers most relevant information, but some topics are left unaddressed. Video is proving valuable to patients.	4	Are additional sources of information provided for patient reference?	Yes/No
The video stands out with exceptional quality and uninterrupted streaming, significantly benefiting patients.	5	Are any areas of uncertainty or debate acknowledged?	Yes/No

Statistical analysis

Statistical Package for the Social Sciences (IBM SPSS Statistics for Windows, IBM Corp., Version 27.0, Armonk, NY) was used to analyze the data collected in the study. Descriptive statistics regarding the distribution of responses to independent variables in the study are presented as numbers and percentages for categorical variables, and as mean, minimum, and maximum for numerical variables.

The compliance of continuous variables with the normal distribution assumption was evaluated with the Kolmogorov-Smirnow test. Kurtosis-skewness coefficients (Kurtosis and Skewness coefficients) were examined to determine the normal distribution. According to the results of Kurtosis and Skewness coefficients, the normal distribution of the data (+2.0/-2.0) was determined.

For pairwise and multiple comparisons, the Chi-square test and Fisher's exact test were used for categorical variables, and independent t-test, one-way analysis of variance (ANOVA) test, or Kruskal-Wallis method were used for quantitative variables.

In comparisons of quantitative variables between more than two groups, post hoc analyses utilized the Bonferroni correction when variances were homogeneous and the Dunnett C test when variances were not homogeneous. The results obtained were considered significant at a 95% confidence interval, with p-values less than 0.05.

## Results

Seventy videos (unreliable group, n=42 (60.0%), reliable group, n=28 (40.0%)) were included in the research. URLs of reliable videos are shown in Table 3. A comparison of reliable and unreliable videos is shown in Table 4.

**Table 3:** URLs of reliable videos

URLs
https://www.youtube.com/watch?v=BpVq2-ftfDc
https://www.youtube.com/watch?v=QhK0p2Lajcs
https://www.youtube.com/watch?v=cZKSOQqZZn0
https://www.youtube.com/watch?v=SEzuoo0h4jc&t=1s
https://www.youtube.com/watch?v=sH_JkuN17LY
https://www.youtube.com/watch?v=nVU1mzt8kl4
https://www.youtube.com/watch?v=-I_G0hxGIXc
https://www.youtube.com/watch?v=RxMb2gabTiQ
https://www.youtube.com/watch?v=lmG-Eio7MRc
https://www.youtube.com/watch?v=CLSdP-vyPGE
https://www.youtube.com/watch?v=jVCNLC8Nh4A
https://www.youtube.com/watch?v=XgCczEyo53w
https://www.youtube.com/watch?v=TvcTwSOKRe4
https://www.youtube.com/watch?v=NoLGqfNvIR4
https://www.youtube.com/watch?v=tjnD9rt4pxY
https://www.youtube.com/watch?v=mXaTAFfEZ_0
https://www.youtube.com/watch?v=UaK6r1cLGzM
https://www.youtube.com/watch?v=TMVEiEmzaXY
https://www.youtube.com/watch?v=xZpAE3TJsfE
https://www.youtube.com/watch?v=fzXEV3qh2x8
https://www.youtube.com/watch?v=Jw5R_jcTtn8
https://www.youtube.com/watch?v=YJpt8nMGhhE
https://www.youtube.com/watch?v=9hW59pvO8tg
https://www.youtube.com/watch?v=kPTniK11Cqg
https://www.youtube.com/watch?v=APreCdL4fMY
https://www.youtube.com/watch?v=PWCdpkQlmTM
https://www.youtube.com/watch?v=5YDMlxTl0k8
https://www.youtube.com/watch?v=06q5JPCOjYo

**Table 4:** Comparison of reliable and unreliable videos MedLat: medial to lateral approach; LatMed: lateral to medial approach

Parameters		Unreliable (n=42)	Reliable (n=28)	p-value
Upload Year (mean, min-max)		4.67 (0-12)	4.43 (0-13)	0.741
Upload Year (mean, min-max)		2019 (2012-2024)	2019 (2011-2024)	0.752
Uploader (n,%)	Society	9 (12.86%)	6 (8.57%)	0.921
	Professional	15 (21.43%)	10 (14.29%)	
	Journal	17 (24.29%)	12 (17.14%)	
Approach (n,%)	MedLat	40 (57.14%)	27 (38.57%)	0.007*
	LatMed	2 (2.86%)	1 (1.43%)	
Audio/Text Description (n, %)	Absence	17 (24.29%)	1 (1.43%)	<0.001*
	Present	25 (35.71%)	27 (38.57%)	
High Definition (HD) (n, %)	HD	22 (31.43%)	14 (20.00%)	0.012*
	720	11 (15.71%)	6 (8.57%)	
	480	7 (10.00%)	7 (10.00%)	
	420	0 (0.00%)	1 (1.43%)	
	548	1 (1.43%)	0 (0.00%)	
	360	1 (1.43%)	0 (0.00%)	
Rank (mean, min-max)		53.38 (4-100)	47.11 (1-99)	0.046*
Duration (mean, min-max)		10.81 (3-31)	12.39 (5-95)	0.041*
Number of Views (mean, min-max)		5053.17 (54-100.000)	6691.04 (392-70843)	0.653
Like (mean, min-max)		53.21 (1-1.000)	64.96 (0-291)	0.716
Dislike (mean, min-max)		-	-	-
Number of Comments (mean, min-max)		1.05 (0-22)	1.47 (0-14)	0.659
Global Quality Scale (GQS) (mean, min-max)		3.50 (1-5)	4.50 (3-5)	<0.001*
Modified DISCERN (mean, min-max)		3.36 (1-5)	4.32 (3-5)	<0.001*

In the research, while the average time from the year the videos were uploaded to the present in the unreliable group was 4.67 (min-max: 0-12) years, in the reliable group it was 4.43 (min-max: 0-13) years in average, and these differences between the groups It was found to be not statistically significant (p=0.752). The average upload year of the videos in both groups was 2019.

In the research, nine (12.86%) of those who uploaded videos in the unreliable group were society, 15 (21.43%) were professional and 17 (24.29%) were journals, while six (12%) of those who uploaded videos in the reliable group were journals. It was observed that six (8.57%) were society, 10 (14.29%) were professional and 12 (17.14%) were journals and these differences between the two groups were not statistically significant (p=0.921).

In the research, in the unreliable group, 40 (57.14%) of the videos were provided by MedLat, while two (2.86%) were provided by LatMed. In the reliable group, 27 (38.57%) of the videos were accessed through MedLat, while one (1.43%) was accessed through LatMed, and these differences between the two groups were found to be statistically significant (p<0.05).

In the research, in the unreliable group, 25 (35.71%) of the videos had audio/text narration, while 17 (24.29%) did not. In addition, in the reliable group, it was observed that 27 (38.57%) had voice/written expressions, while one (1.43%) did not, and these differences between both groups were found to be significant (p<0.001).

In the study, the HD resolutions of the patients in the unreliable group were at most 22 (31.43%) HD and 11 (15.71%) in the 720 band, while the HD resolutions of the patients in the reliable group were at most 14 (20.0%) HD and six (8.57%) had resolution in the 720 band, and these differences between both groups in terms of HD resolution were statistically significant (p<0.05).

In the study, it was observed that the average ranking of the videos in the unreliable group was 53.38 (4-100), while the average ranking of the videos in the reliable group was 47.11 (1-99) and these differences between the two groups were significant (p<0.05).

In the study, it was determined that the average duration of the videos in the unreliable group was 10.81 minutes, while the average duration of the videos in the reliable group was 12.39 minutes and these differences between the two groups were significant (p<0.05).

In the study, the average number of views of the videos in the unreliable group was 5053.17 (54-100,000), while the average number of views of the videos in the reliable group was 6691.04 (392-70843) and it was found that these differences between the two groups were not significant (p=0.653).

In the research, it was found that the average number of likes of the videos in the unreliable group was 53.21 (1-1,000), while the average number of likes of the videos in the reliable group was 64.96 (0-291) and these differences between the two groups were not significant (p=0.716).

In addition, while the average number of comments made to the videos in the unreliable group was 1.05 (0-22), the average number of comments made to the videos in the reliable group was 1.47 (0-14) and these differences between the two groups were not significant. It was found that it was not (p=0.659).

In the research, the average GQS value of the videos in the unreliable group was 3.50 (1-5), while the average GQS value of the videos in the reliable group was 4.50 (3-5), and both were observed that these differences between the groups were significant (p<0.001).

In the research, it was observed that the average modified DISCERN value of the videos in the unreliable group was 3.36 (1-5), while the average modified DISCERN value of the videos in the reliable group was 4.32 (3-5) and these differences between the two groups were significant (p<0.001).

## Discussion

YouTube videos about LRHCME were reviewed for accuracy and quality. The results of the current study indicate that LRHCME videos on YouTube are insufficient for surgical training. It is thought that with the increase in internet use in recent years, obtaining information by watching videos has increased [15]. Due to the variety of uploaded videos, YouTube is a non-negligible platform for gaining knowledge. However, since everyone is accessible and videos can be uploaded by anyone, the quality, accuracy, and educational value of the videos vary [16]. It can be said that the reliability of the videos uploaded to YouTube is low because they are not evaluated by a reviewer. However, although the videos were uploaded by health professionals, society, and journals, they were largely unreliable and there were no significant differences between these uploaders. In the study conducted by Ferhatoğlu et al., the videos uploaded to WebSurg were below the expected quality, even though they were uploaded by health professionals [17].

To make money and advertise on social media, the popularity of videos rather than their accuracy comes to the fore [18]. In fact, recently, users have turned to short videos that are edited for high viewing rates, provide instant information, and whose accuracy is unknown [19]. Viewers now turn to shorter videos. Short videos are appealing because they need minimal time investment from viewers [20]. We are not talking about short one-minute videos for our work. However, the audience now tends towards shorter videos. In our study, it was found that the video duration was significantly shorter in unreliable videos.

In our study, we found that reliable videos were ranked higher when searched according to default settings. Therefore, video screening criteria can provide relatively reliable results compared to default settings.

We wanted to see which of the approach techniques was more common. We found that the MedLat approach was used in LRHCME in most of the videos. However, both approaches are reliable and they are not superior to each other [21]. However, in the MedLat approach, it is believed that it is easier to cut the vascular pedicle before the surgical field becomes contaminated, then mobilize the mesentery towards the abdominal wall, and finally release the colon through Toldt's fascia [22]. Additionally, a clear anatomical representation for video presentation is more possible with the MedLat approach. For these reasons, the MedLat approach is more common.

Since the steps of preoperative preparation, setup of laparoscopy, closure of the extraction area and port areas, and completion of the operation are not shown in almost any video, we evaluated them over 16 steps. All videos were evaluated by two experienced surgeons. The most frequently neglected step that made the videos unreliable was not showing trocar placement.

HD resolution quality, modified DISCERN, GQS, and the audio/text comment ratio were higher in reliable videos. GQS was used to measure the educational value of videos by Bernard et al. [23]. Modified DISCERN is also used to evaluate videos' reliability and quality dimensions [24,25]. It is not unexpected that both were found higher in reliable videos. Other studies evaluated DISCERN and GQS scores between healthcare professional-uploaded and nonprofessional-uploaded videos [26-28]. In the study conducted by Mutlu et al., GQS and DISCERN scores were lower in videos uploaded by non-professionals [29]. However, in our study, all of the videos were uploaded by healthcare professionals, journals, and medical societies.

Study limitations

There are some limitations in our study. All searches were performed within a certain period based on relevance in the YouTube search engine. Different time periods may affect search results, and the quality, relevance, and engagement level may vary between users. Two experienced surgeons performed our evaluations. However, assessments made by different experts may produce different results.

## Conclusions

In our study, we examined the quality of information shared in YouTube videos of LRHCME, a complex oncological surgical procedure. Based on the Delphi consensus, videos were divided into reliable and unreliable. The rate of unreliable videos was quite high and the information shared was insufficient. Therefore, YouTube alone is not a reliable source for learning LRHCME. YouTube's popularity-focused metrics like likes and views should not be viewed as an indicator of trustworthiness. YouTube needs to improve its rankings and recommendation system to promote more trustworthy content.
